# Stomatal Closure Sets in Motion Long-Term Strategies of Plant Defense Against Microbial Pathogens

**DOI:** 10.3389/fpls.2021.761952

**Published:** 2021-09-27

**Authors:** Shashibhushan Gahir, Pulimamidi Bharath, Agepati S. Raghavendra

**Affiliations:** Department of Plant Sciences, School of Life Sciences, University of Hyderabad, Hyderabad, India

**Keywords:** guard cells, innate immunity, peroxisomes, photorespiration, secondary messengers, transpiration

## Introduction

Stomata are the main gateways for the entry of microbial pathogens into leaves (Melotto et al., 2008). However, some try to use hydathodes (Hugouvieux et al., 1998) or breach the cuticle (Grimmer et al., 2012). Stomatal closure, therefore, is an effective measure to restrict pathogen entry and provide the plants an innate immunity (Melotto et al., 2008, 2017; Sawinski et al., 2013; Bharath et al., 2021). Stomata open when the guard cells are turgid and close when guard cells are flaccid (Willmer and Fricker, 1996). Whenever plants are exposed to stress, the guard cells sense and respond by a series of steps that include the production of ROS and NO followed by a rise in Ca^2+^ and the modulation of ion channels. These events promote the efflux of cations and anions from guard cells. As a result, guard cells lose turgor leading to stomatal closure (Arnaud and Hwang, 2015; Agurla et al., 2018; Saito and Uozumi, 2019; Hsu et al., 2021).

The reopening of stomata is usually slower than the closure, ensuring that the leaves conserve water for an extended period. For example, abscisic acid (ABA)-induced stomatal closure in the epidermis took about 30 min (and in leaf 3 h). In contrast, recovery took 1–6 days, implying short-term and long-term effects on stomata (Liang and Zhang, 1999). Recently, we pointed out that the stomatal closure by ABA was an essential component of plant adaptation to stress factors (Bharath et al., 2021). This article proposes that the initial stomatal closure response triggers many defensive strategies to fight the pathogens. We describe the follow-up of events limiting pathogen spread and emphasize stomata's role in ensuring plants' long-term adaptation against microbes.

### Stomatal Closure: An Immediate Barrier of Microbial Entry

Stomatal closure was a typical response against microbial attack (Arnaud and Hwang, 2015; Melotto et al., 2017; Agurla et al., 2018). The process of stomatal closure is initiated by sensing the abiotic (e.g., drought, chilling, and UV-B) or biotic stress (pathogens and insects) components (Agurla et al., 2018). Most microbial pathogens produce pathogen-or microbe-or damage-associated molecular patterns (PAMPs/MAMPs/DAMPs), perceived by pattern recognition receptors (PRRs) present on the plant plasma membrane. Upon perception, plants activate a defense response called pattern-triggered immunity (PTI). When pathogens attempt to overcome PTI, plants trigger effector-triggered immunity (Cui et al., 2015; Nguyen et al., 2021). Bacterial elicitors that trigger stomatal closure include flagellin22 (flg22), lipopolysaccharide, and other elicitor peptides, such as, elf26 (Melotto et al., 2008, 2017; Arnaud and Hwang, 2015). Fungal elicitors, such as, chitin oligosaccharide and chitosan, also induced defense responses in plants (Ye et al., 2020).

Guard cells perceive hormones (e.g., ABA) or elicitors (flg22) by their respective receptors. Upon binding to ABA or flg22, the receptor kinases (e.g., *open stomata 1* or *botrytis-induced kinase 1*) activate RbohD/F and stimulate reactive oxygen species (ROS) production during stomatal closure. However, the role of RBOHD in resistance against pathogens, particularly during pre-invasive stage is not clear. The elevated ROS, in turn, trigger a rise in nitric oxide (NO) and Ca^2+^. The interaction of these secondary messengers (ROS/NO/Ca^2+^) regulates the downstream components in guard cells. Both NO and Ca^2+^ (via Ca^2+^-dependent protein kinases) promote the ion efflux by activating K^+^ out, SLAC1, and SLAH3 channels and at the same time inhibit the K-influx channel (Arnaud and Hwang, 2015; Agurla et al., 2018; Kohli et al., 2019; Sun et al., 2019). Similarly, ROS and Ca^2+^ activate Ca^2+^ influx and increase cytosolic Ca^2+^ levels (Klüsener et al., 2002). The elevated ROS, NO, Ca^2+^ and H_2_S provide an extended pathogen resistance (Gahir et al., 2020; Liu and Xue, 2021). Cytosolic pH is another secondary messenger that preceded the production of ROS and NO in guard cells, but the exact mechanism is ambiguous (Gonugunta et al., 2009; Bharath et al., 2021). It is necessary to study if such changes in pHcyt can modulate the pathogen resistance as well.

### Stomatal Closure Associated With the Modulation of Plant Hormones

Stomatal closure during drought or microbial infection was associated with an increase in plant hormones. Salicylic acid (SA), ABA, methyl jasmonate (MJ), and ethylene (ET) accumulate when microbes attack plants. The concerted action of these hormones causes stomatal closure and induces systemic resistance (Gimenez-Ibanez et al., 2016; van Butselaar and Van den Ackerveken, 2020; Bharath et al., 2021). The modulated hormonal status provides long-term protection to plants against biotic and abiotic stress (Described below).

## Discussion

### Closure Triggers a Network of Long-Term Events to Ensure the Protection

Stomatal closure in response to microbial infection is an immediate physical measure to prevent microbial entry. However, such closure has long-term effects, such as, a marked decrease in the intercellular CO_2_ of leaves, a reduction in photosynthetic carbon assimilation, and an elevation in photo respiratory activity. The reduction in transpiration can cause mineral deficiency in leaves. We describe below the consequences of these events and a few associated components.

#### Decrease in Photosynthesis and Increase in Photorespiration and Peroxisomal Population

When stomata close, the intercellular CO_2_ is lowered, and transpiration decreased, raising the leaf temperature. Both these factors enhance photorespiration. The increase in photorespiration occurred under conditions of biotic (microbial infection) or abiotic stress (drought) (Lal et al., 1996; Pascual et al., 2010; Voss et al., 2013; Vo et al., 2021). Even fluctuations in transpiration triggered an increase in photorespiration (Furutani et al., 2020). The enhanced photorespiration and the associated rise in H_2_O_2_ could confer disease resistance (Taler et al., 2004; Kubo, 2013; Sørhagen et al., 2013). Further, glycolate, glyoxylate, and glycine, being pathway intermediates, accumulate. Glycolate and glyoxylate are toxic to living cells and can double up as antimicrobial compounds. Glycine is the precursor of glutathione, an essential anti-oxidant in plant cells. Photo respiratory enzymes/metabolites mediated the plant defense during tomato-*Pseudomonas syringae* interactions (Ahammed et al., 2018). Thus, photo respiratory metabolism could help to resist pathogens.

The enhanced photorespiration was often associated with an increase in the peroxisomal population in leaf cells (Chen et al., 2016). Peroxisomal ROS could protect against plant pathogens (Sørhagen et al., 2013). Besides ROS, other components of peroxisomes, namely NO, Ca^2+^, and polyamines (PA), upregulated the genes involved in SA signaling and PA catabolism, reinforcing plant defense responses (Chen et al., 2016; Wang et al., 2019).

#### Stomatal Closure Lowers Leaf Sugars

Stomatal closure, whether due to pathogen attack or drought, causes reduced CO_2_ assimilation and decreased carbon partitioning into sucrose and starch (Wang et al., 2016; Haider et al., 2017). The pathogens required sugars for growth and infection (Solomon et al., 2003; Scharte et al., 2005; Chang et al., 2017). If sufficient sucrose is not available, the extent of proliferation would be restricted (Huai et al., 2020). Therefore, the deficiency in sugar availability lead to decreased fungal growth (Bezrutczyk et al., 2018).

#### Reduced Transpiration Creates Mineral Deficiency

Transpiration is a prerequisite for long-distance transport of minerals (Ruiz and Romero, 2002). A deficiency of minerals would occur when stomata are closed. There was a positive relationship between the transpiration rate and mineral content of sunflower (*Helianthus annuus*) and maize leaves (Tanner and Beevers, 2001; Shrestha et al., 2021). Since microbial spread and multiplication within leaves depend on macronutrients/micronutrients, the mineral deficiency could affect microbial growth and enhance pathogen tolerance (Fernández-Escobar, 2019). The N-status of leaves modulated defense-related hormones, NO content, and then genes (Sun et al., 2020). The deficiency of N increased the levels of phenolics and restricted the spread of powdery mildew (Bavaresco and Eibach, 1987). A similar situation under K^+^-deficiency was reported with leaf spot, caused by *Helminthosporium cynodontis* (Richardson and Croughan, 1989). Other examples of mineral deficiency that favor pathogen resistance were zinc (Cabot et al., 2019) and iron (Trapet et al., 2021). Readers can find a detailed description of the dual role of the macro-and micronutrients for the infection by bacterial and fungal pathogens elsewhere (Huber et al., 2012).

#### Continuing Effects of Secondary Messengers, Plant Hormones, and Secondary Metabolites

The secondary messengers produced during stomatal closure can continue to protect plants. For example, the combination of ROS/NO/Ca^2+^ was quite effective in limiting the spread and multiplication of microbes within the leaf. These secondary messengers trigger hypersensitive response (HR), synthesis of pathogenesis-related (PR) proteins, and programmed cell death (PCD) (Serrano et al., 2015; Marcec et al., 2019). Besides NO, H_2_S produced during stomatal closure could confer pathogen resistance (Vojtovič et al., 2020). It is possible that these components ROS/NO/Ca^2+^ can also induce priming effect individually or in combination.

When plants were infected by pathogens, the leaves responded by modulating the hormones, which interacted with each other to impart a long-lasting response. Plant hormones (e.g., SA, methyl salicylate, MJ) and even PAs could induce systemic resistance (Bürger and Chory, 2019; Chen et al., 2019; Seifi et al., 2019; Yuan et al., 2019). These hormones (ABA/MJ/SA) primed the plant tissue to stand against pathogens (Agostini et al., 2019; Feng et al., 2020). These observations open up several exciting lines of work for further research.

Several secondary metabolites produced by the plants are prominently associated with protection against bacterial, fungal, and viral attacks. The elevated levels of H_2_O_2_, NO, and Ca^2+^ induced accumulation of secondary metabolites like wax, callose, alkaloids, flavonoids, phenols, and PAs, reinforcing the protection against infection (Walters, 2003; Luna et al., 2011; Zaynab et al., 2018; Lewandowska et al., 2020). The PAs also prime the plants against *Botrytis* (Janse van Rensburg et al., 2021). Similarly, allyl isothiocyanate (AITC) keeps microbes like *P. syringae* out by inducing stomatal closure (Bednarek, 2012).

## Conclusion and Future Perspective

**Figure 1:**
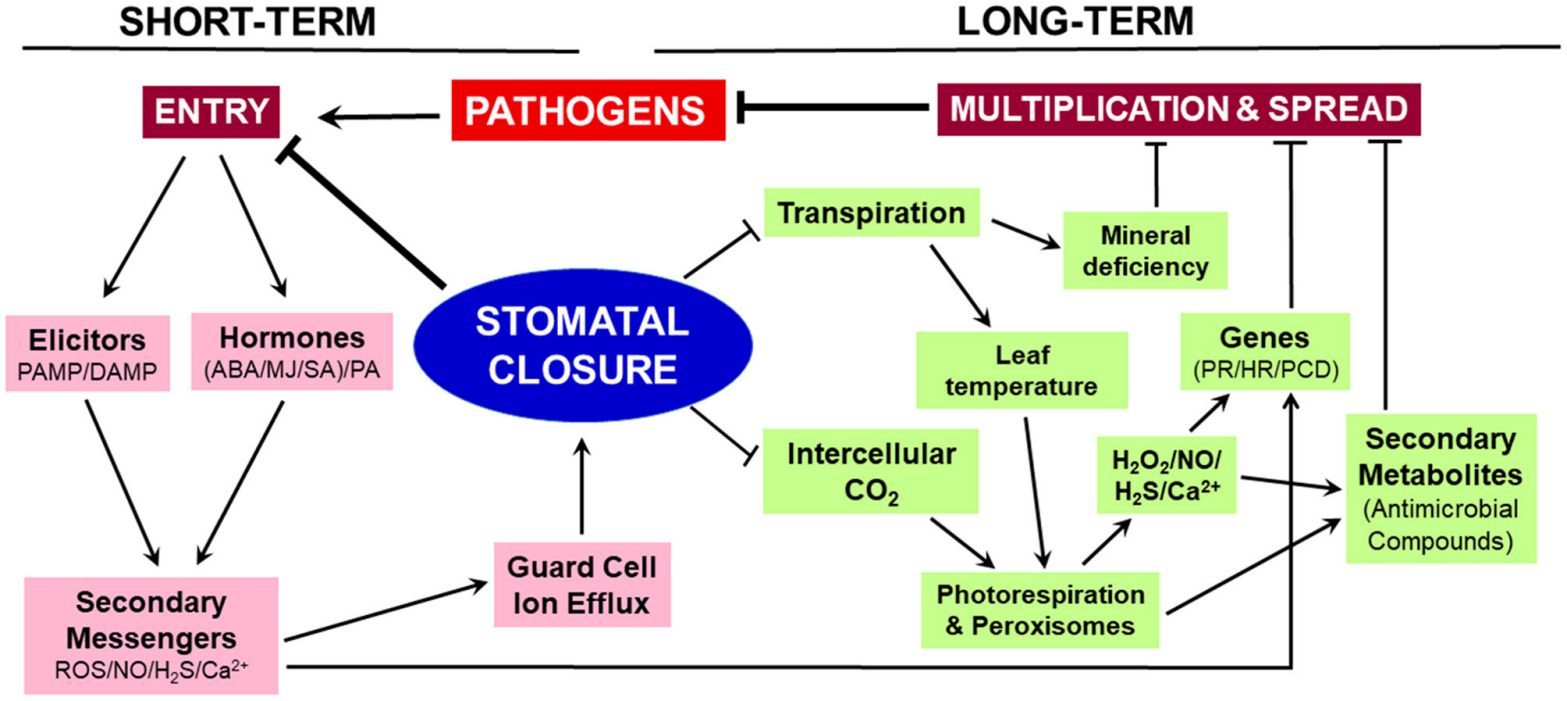
Schematic representation of events associated with stomatal closure. The closure restricts microbial entry during the short term, while the events initiated during closure contribute to long-term adaptation against microbes. On sensing the microbial elicitor molecules, the guard cells trigger a rise in ROS, NO, Ca^2+^, and H_2_S. These secondary messengers cause ion-efflux from guard cells and stomatal closure. As a consequence of closure, the entry of CO_2_ and transpirational H_2_O loss are restricted, leading to increased photorespiration, decreased leaf sugars, and mineral deficiency. All these events help in restricting microbial multiplication, spread, and growth. The disturbed hormonal status and reactive molecules ensure continued protection by upregulating PR/HR/PCD genes and promoting the levels of antimicrobial secondary metabolites. Arrow represents an increase and ⊣ indicates a decrease.

Stomatal closure erects a physical barrier providing immediate relief against the entry of microbial pathogens into leaves while decreasing the rates of photosynthesis and transpiration. The closure has long-term consequences (Figure 1). The restricted CO_2_ supply to the mesophyll cells lowers the rate of photosynthesis, stimulates photorespiration and associated H_2_O_2_ production. The elevated levels of H_2_O_2_, along with NO, H_2_S, and Ca^2+^, can upregulate genes involved in HR, PR, and PCD to prevent the spread of pathogens within the leaf. These reactive molecules also promote the accumulation of antimicrobial secondary metabolites. Parallelly, reduced transpiration creates mineral deficiency and limits microbial growth. We suggest that stomatal closure is a trigger to set off long-term events involved in prolonged plant disease resistance.

We know that stomatal closure may not be a universal mechanism to fight the microbial attack, e.g., root or stem pathogens. But several pathogens are air-borne and land on leaves (Melotto et al., 2008; Zeng et al., 2010). Plant-microbe interactions are not unilateral since the pathogens try to reopen stomata using compounds, such as, coronatine (Arnaud and Hwang, 2015). Further work is needed to understand the implications of stomatal closure on the antagonizing responses by the pathogens. Peroxisomal H_2_O_2_ limits microbial growth, but there are instances when microbes use peroxisomes to their advantage (Kubo, 2013). An improved understanding of peroxisomes and manipulation through biotechnological techniques could open up possibilities of designing plants for long-term adaptation to stress conditions. We believe that stomatal guard cells are ideal for studying plants' short-and long-term responses to challenging stress situations. Stomatal closure can be exploited to improve crop growth and grain yield under environmental stress conditions. In crops such as, wheat and rice, reduced water requirement due to stomatal closure was used as one of the physiological traits in crop breeding (Park et al., 2020; Paul et al., 2020). Further studies on the long-term effects of stomatal closure can be translated into additional field applications.

## Author Contributions

AR conceptualized the idea, prepared the outline, and edited the final version. SG, PB, and AR pooled relevant literature, wrote the manuscript, and approved the final manuscript. All authors contributed to the article and approved the submitted version.

## Funding

The stomatal work in our laboratory was supported by a grant (to AR) of the Council of Scientific and Industrial Research [No. 38 (1404)/15/EMR-II]. SG was supported by a Senior Research Fellowship from University Grant Commission, New Delhi. PB was supported partially by a University of Hyderabad BBL fellowship.

## Conflict of Interest

The authors declare that the research was conducted in the absence of any commercial or financial relationships that could be construed as a potential conflict of interest.

## Publisher's Note

All claims expressed in this article are solely those of the authors and do not necessarily represent those of their affiliated organizations, or those of the publisher, the editors and the reviewers. Any product that may be evaluated in this article, or claim that may be made by its manufacturer, is not guaranteed or endorsed by the publisher.
